# Identification and Disruption of Sperm-Specific Angiotensin Converting Enzyme-3 (ACE3) in Mouse

**DOI:** 10.1371/journal.pone.0010301

**Published:** 2010-04-22

**Authors:** Naokazu Inoue, Tatsuya Kasahara, Masahito Ikawa, Masaru Okabe

**Affiliations:** Research Institute for Microbial Diseases, Osaka University, Osaka, Japan; Sun Yat-Sen University, China

## Abstract

**Background:**

IZUMO1 is the only sperm protein which is proven to be essential for sperm-egg fusion. However, the IZUMO1 is a structurally simple protein with single Ig domain and seems not to include either a “fusogenic peptide” or a fusion machinery domain. This led us to assume the existence of an IZUMO1-interacting protein(s) which makes a functional fusion machine interacting with IZUMO1.

**Methodology/Principal Findings:**

We produced a transgenic mouse line which expresses His-tagged IZUMO1 in the *Izumo1* ^−/−^ genetic background. After solubilization of sperm membranes, we purified His-tagged IZUMO1 using anti-His affinity chromatography and found a protein that interacts with IZUMO1. After being separated on SDS-PAGE gel, the IZUMO1-interacting protein was subjected to LC-MS/MS analysis and from the partial fragments, we identified the protein as ACE3. We raised the antibody against ACE3 and found that ACE3 is localized on the acrosomal cap area as in the case of IZUMO1. However, ACE3 disappeared from sperm after acrosome reaction while IZUMO1 remained on sperm. In order to investigate the role of ACE3 *in vivo*, we generated *Ace3*-deficient mice by homologous recombination and examined the fertilizing ability of the males. Unexpectedly, the male mice showed no defect in fertilizing ability in *in vivo* or in an *in vitro* fertilization system.

**Conclusions/Significance:**

We identified an IZUMO1-interacting protein in sperm, which we identified as testis specific ACE homologue ACE3. We produced an *Ace3* disrupted mouse line, and found the localization of IZUMO1 spread in a little wider area on sperm, but the elimination of ACE3 did not result in a loss of sperm fertilizing ability, differing from the case of ACE disruption.

## Introduction

In mammals, the sperm deposited in the female reproductive tract start their journey to the ovulated eggs, residing in the ampullar portion of the oviduct. Sperm must undergo a physiological change called “capacitation” and a morphological change called “acrosome reaction”. During acrosome reaction, sperm shed the plasma membrane in their acrosomal cap area and newly expose the inner- acrosomal membrane. It is known that only acrosome reacted sperm have an ability to fuse with eggs. Despite the biological importance of sperm-egg fusion in fertilization, the molecular mechanism of this step remains virtually unknown.

Recently, experiments using gene-manipulated animals unveiled two proteins as essential factors in sperm-egg fusion in mouse. First, a tetraspanin family CD9 was serendipitously and simultaneously found to be essential for eggs to fuse with sperm in research from 3 independent laboratories [1], [2], [3]. Then we identified a novel sperm-specific protein, IZUMO1, as an essential factor for sperm to fuse with eggs [4]. IZUMO1 is a transmembrane protein with an extracellular region, a single transmembrane region and a short cytoplasmic tail. A computer program domain search revealed no structural motifs in IZUMO1 except one immunoglobulin (Ig)-like domain in the extracellular region. Ellerman et al. reported that there is a dimerization site upstream of an Ig-like domain by western blot analysis under mildly denaturing conditions using different recombinant IZUMO1constructs [5]. However, they failed to demonstrate binding of a recombinant IZUMO1 peptides with the eggs. Since IZUMO1 has a simple structure compared to other fusion proteins [6] with no “fusogenic” peptide or “SNARE” like structure in it, we entertained the possibility that IZUMO1 is one of the components that form a fusion competent structure on sperm.

Based on these observations, we tried to find an IZUMO1-interacting protein. To make the purification of the interacting protein easier, we produced His-tagged IZUMO1 expressing transgenic mouse sperm. This mouse line was crossed to *Izumo1* ^−/−^ genetic background, giving rise to a line which produces no wild-type IZUMO1 but only His-tagged IZUMO1. This strategy was combined with a purification method using anti-His magnetic beads and the putative IZUMO1-interacting protein was subjected to LC-MS/MS analysis. Interestingly, the protein was identified as an angiotensin converting enzyme (ACE) homologue ACE3. ACE3 has been reported as a novel homologue of testis-specific ACE (tACE), but only at mRNA level [7]. We demonstrated the exclusive expression of ACE3 protein in testis and sperm, and we named it testis-specific ACE3 (tACE3). Although the function of tACE3 is totally unknown, its homologue somatic ACE (sACE) is a protein well-known in the regulation of blood pressure. However, the function of ACE is not limited to cardiovascular homeostasis, but includes a totally a different function which allows sperm to migrate into the oviduct and bind to the zona pellucida that surrounds the egg [8]. Therefore, when ACE was disrupted by homologous recombination, the male mice became infertile. After transgenic rescue experiments, it was clarified that tACE (transcribed from testis specific promoter residing 12th intron of sACE) rather than sACE, actually functions in male fertility [9]. In this context, it is very interesting that the purified putative IZUMO1-interacting protein was the tACE3. In the present paper, in order to elucidate the physiological role of tACE3, we produced an *Ace3* disrupted mouse line and analyzed the fertilizing ability of these mice both *in vitro* and *in vivo*.

## Results

### Identification of IZUMO1-interacting protein

**Figure 1:**
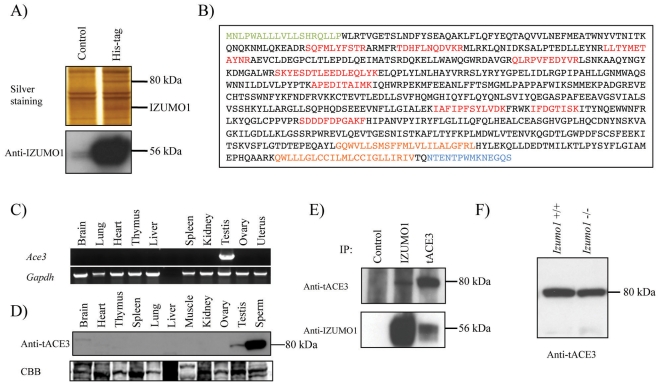
Identification and characterization of the IZUMO1-interacting protein. (A) The purified IZUMO1 protein complex was separated by SDS-PAGE and stained with silver. Two specific 80-kDa and 56-kDa bands appeared corresponding to tACE3 and IZUMO1, respectively. Proteins from wild-type sperm were used as controls. (B) Amino-acid sequences of mouse tACE3. The peptide sequences obtained by LC-MS/MS are shown in red. The putative signal peptide and transmembrane region are shown in green and orange, respectively. Antigen for producing tACE3-specific antibody is shown in blue. (C) Reverse transcription-PCR of *Ace3* (upper panel) and *Gapdh* (control; lower panel) from 10 mouse cDNAs. (D) tACE3 was detected exclusively in testis and sperm by western blotting. All solubilized proteins were loaded at 30 µg on each lane and detected by 1 µg/ml anti-tACE3 antibody. (E) Interaction between IZUMO1 and tACE3. Total sperm proteins (∼100 µg) prepared from wild-type male mice were immunoprecipitated with anti-IZUMO1 or anti-tACE3 antibodies and blotted with anti-tACE3 or anti-IZUMO1 antibodies, respectively. (F) tACE3 protein exists in *Izumo1*-deficient sperm.

To identify IZUMO1-interacting proteins, we used acrosome intact sperm lysate prepared from a transgenic mouse line which had IZUMO1-His in the *Izumo1* ^−/−^ background [10]. These transgenic lines were fertile, suggesting that the His-tagged IZUMO1 protein is functionally normal. This His-tagged IZUMO1 protein was purified by the Miltenyi MACS beads system using anti-His microbeads. The proteins eluted with boiled SDS-PAGE sample buffer were separated by SDS-PAGE. Proteins from wild-type epididymal sperm were used as negative control. Two specific bands were detected by silver staining (Figure 1A). The bands from a purification using ∼100 mg of protein (10 male mice) were silver stained, excised, trypsinized, and subjected to LC/MS/MS analysis. The peptides from the 56-kDa band were identified as IZUMO1 protein. We also confirmed IZUMO1 protein by western blot analysis with IZUMO1 antibody (Figure 1A, lower panel). From the 9 peptides in the 80-kDa band, a functionally unknown protein (ACE3: angiotensin I converting enzyme 3) was identified (Figure 1B).

### Characterization of ACE3

*Ace3* mRNA has been described by Rella et al. [7]. They reported that the *Ace3* gene is located on chromosome 11 downstream of the *Ace* gene, and that *Ace3* mRNA is expressed in heart and testis. In mouse, the predicted protein sequence for ACE3 has two hydrophobic regions at the C-terminus but no N-terminal signal peptide [7]. After RT-PCR using total RNA prepared from testis, we examined *Ace3* mRNA alignment by sequencing. As a result, we found a new transcript of *Ace3* mRNA (NCBI accession number: AB531024) consisted with 2214 nucleotides which encode a predicted protein of 737 amino acids (Figure 1B). The predicted amino acid sequence from the alternative form of *Ace3* transcript has a signal peptide (Figure 1B, shown in green) differing from the originally reported ACE3. The original form of Ace3 matched completely to the amino acid sequence from the 31st Asp to the C-terminal amino-acid in the alternative ACE3 we cloned in this experiment. Among the ten adult tissues examined, in our hands, only testis had detectable expression of *Ace3* mRNA (Figure 1C). To check the existence of ACE3 protein, we developed a polyclonal antibody against amino acids 724–737 (Figure 1B, shown in blue) of the ACE3 protein. The ACE3 antibody specifically recognized a single major 80 kDa-band exclusively in testis and sperm (Figure 1D). We refer to the ACE3 as tACE3 (testis-specific ACE3) hereafter. Anti-tACE3 antibody immunoprecipitated IZUMO1 from sperm protein, further confirming the interaction of the two proteins (Figure 1E). We also examined if the disruption of *Izumo1* induced the aberrant expression of tACE3. However, the tACE3 was detected in similar amounts on Izumo1^−/−^ and wild-type sperm (Figure 1F).

### Subcellular localization of tACE3

**Figure 2:**
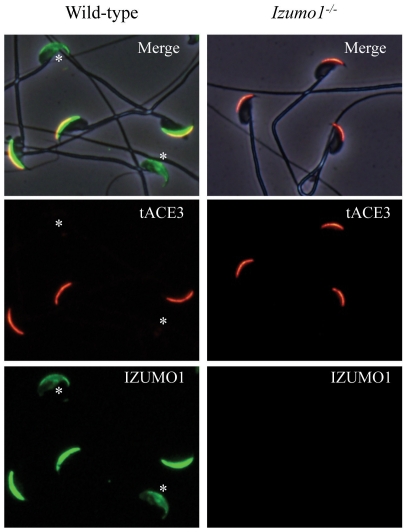
Subcellular localization of tACE3 protein in mature sperm. Incubated sperm (left) and *Izumo1* knockout sperm (right) were prepared from cauda epididymis. They were stained with 4 µg/ml anti-tACE3 (red) and anti-IZUMO1 (green) antibodies. The anti-tACE3 antibody stained acrosome-intact sperm head, but not reacted to acrosome-reacted sperm (asterisk).

We performed an immunofluorescent staining experiment to analyze the subcellular localization of tACE3. The tACE3 in fresh mouse sperm were brightly stained in the acrosomal cap area in 100% EtOH-fixed sperm similar to SP56 and ACROSIN [11], [12] (Figure 2). In order to detect the localization of tACE3 after acrosome reaction, we incubated sperm in TYH medium for 2 h, fixed, and incubated with antibodies. To differentiate the acrosome intact and reacted sperm, we double stained the sperm with anti-IZUMO1 antibody, because the anti-IZUMO1 antibody stains acrosomal cap and the entire head regions in acrosome intact and acrosome reacted sperm, respectively. The tACE3 was detected only in the acrosomal cap area of acrosome intact sperm co-localizing with IZUMO1, but disappeared from acrosome reacted sperm. This indicates that tACE3 protein is released, along with fused acrosomal vesicle membranes, from the sperm head during spontaneous acrosome reaction (Figure 2). The localization of tACE3 was examined in *Izumo1* ^−/−^ sperm, but the staining pattern remained normal, resembling that of wild-type sperm (Figure 2).

### Generation of tAce3-deficient mice

**Figure 3:**
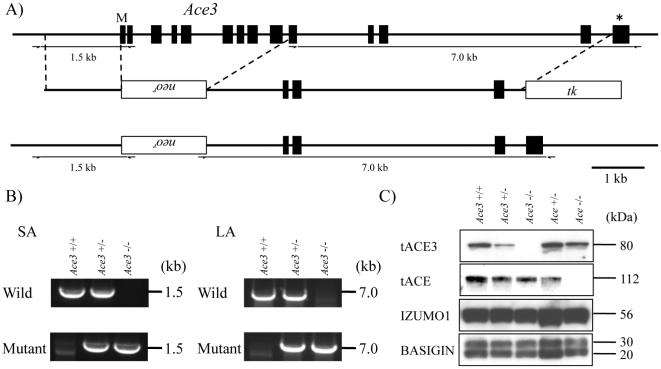
Targeted disruption of *Ace3* gene. (A) Complete structures of the wild-type mouse *Ace3 allele*. Exons and introns are represented by boxes and horizontal lines, respectively. For the targeted disruption of mouse *Ace3* allele, the first 9 exons (closed boxes) were replaced by the neomycin-resistant gene (Neo^r^). A herpes simplex virus thymidine kinase gene (tk) was introduced into the targeting construct for negative control. (B) Genotyping of tail tip DNA by PCR amplification with primers indicated in the figure. (C) Western blotting analysis of sperm lysates from wild-type, *Ace3* ^+/−^, *Ace3* ^−/−^, *Ace* ^+/−^ and *Ace* ^−/−^ mice.

To clarify the physiological role of tACE3 *in vivo*, we generated *Ace3*-deficient mice by homologous recombination. Previous reports showed that the mouse *Ace3* gene contains 13 exons, and is located 5.5 kb downstream of the ACE gene [7]. However, the *Ace3* gene consisted 14 exons (Figure 3A). Thus, the *Ace3* must be transcribed from an alternative promoter. The targeting vector was designed to remove the 1st to the 9th exon of *Ace3* (which corresponds to the 1st to the 8th exon of previous *Ace3*) (Figure 3A) and was electroporated into 129Sv ES cells after linearization. Potentially targeted ES cell clones were separated by positive-negative selection with G418 and acyclovir. ES clones that had correctly targeted the *Ace3* allele were screened by PCR on both ends of the targeting vector (Figure 3B). After obtaining chimeric mice that transferred mutant allele to the next generation, the heterozygous mutant mice were established. When they were crossed, the inheritance of the *Ace3* disrupted allele yielded the expected Mendelian ratios. The *Ace3* ^−/−^ mice exhibited normal development and grew up as healthy adults. The disappearance of tACE3 in *Ace3* ^−/−^ mice was confirmed by western blot analysis (Figure 3C). To examine the effect of tACE3 disappearance on the expression of IZUMO1 protein, we carried out western blot analysis using epididymal sperm. The amounts of IZUMO1 were not decreased in *Ace3* ^−/−^ sperm (Figure 3C).

### Fertilizing ability of tAce3-deficient sperm

**Figure 4:**
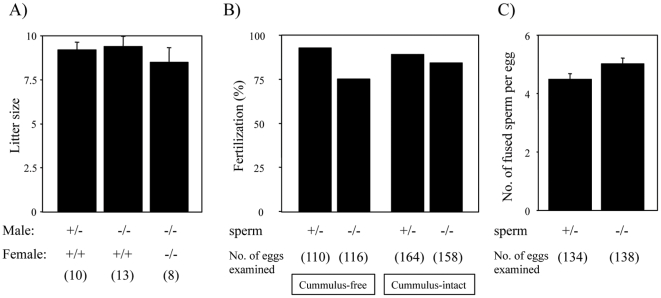
Fertility analysis of *Ace3* ^−/−^ mice. (A) Fecundity of *Ace3* ^+/−^ and *Ace3* ^−/−^ males and *Ace3* ^−/−^ females. The numbers in parentheses indicate the numbers of mating pairs. Values are presented as mean ± standard error of mean (SEM). (B) *in vitro* fertilization of sperm from *Ace3* ^+/−^ and *Ace3* ^−/−^ mice using cumulus-free and –intact eggs (n = 5). (C) Comparison of the fusing ability of *Ace3* ^+/−^ and *Ace3* ^−/−^ sperm. Average numbers of fused sperm observed 30 minutes after insemination (n = 5). Values are presented as mean ± SEM.

*Ace3* ^−/−^ mice were mated with mice in various combinations but we could not detect any sign of infertility (Figure 4A). When sperm were prepared from *Ace3* ^−/−^ male and observed, we found no difference in their sperm number or the motility compared to wild-type mice (data not shown). We then analyzed the fertilizing ability of *Ace3* ^−/−^ sperm in the *in vitro* fertilization system, where in some cases the defect can be detected [13]. However, the *Ace3* ^−/−^ sperm showed normal fertilizing ability relative to *Ace3* ^+/−^ sperm under the in *in vitro* fertilization systems using both cumulus-intact and -free eggs (Figure 4B). The sperm-egg fusion assay using zona-free eggs also indicated that *Ace3* ^−/−^ sperm possessed normal fusion ability with eggs (Figure 4C). These results suggest that tACE3 is not an essential element for fertilizing ability of sperm.

### Effect of Ace3 disruption in localization of IZUMO1

**Figure 5:**
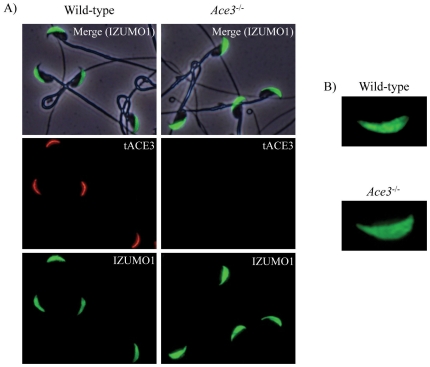
Immnolocalization of IZUMO1 protein in *Ace3* ^−/−^ sperm. Wild-type and *Ace3* ^−/−^ sperm were immunostained with 4 µg/ml anti-tACE3 (red) and anti-IZUMO1 (green) antibodies (A). The staining pattern of IZUMO1 in *Ace3* ^−/−^ sperm showed a significantly broader staining area than that of wild-type sperm (B).

Finally, we examined the effect of tACE3 disruption on the localization of IZUMO1 antigen on sperm by indirect immunofluorescent staining. In this experiment, we could detect a slightly deformed localization of IZUMO1 in the acrosome cap region before acrosome reaction. The localization of IZUMO1 in *Ace3* ^−/−^ sperm appeared to be broader than that examined in wild-type sperm (Figure 5A–B). The reason for this difference is not clear but this slight variation was not critical for sperm to maintain their fertilizing ability.

## Discussion

Although fertilization is a very important biological phenomenon, the mechanism of sperm-egg interaction is not well understood. There are many factors that have been reported to be “important” for fertilization; however, most of them emerged as inessential following gene knockout experiments [14], [15]. As of today, IZUMO1 is the only sperm protein which is proven to be essential for sperm-egg fusion. However, the protein structure of IZUMO1 is rather simple with only one Ig domain in its outer surface area. There seems to be no “fusogenic peptide” domain or domains reminiscent of other fusion proteins reported in viruses or other systems in IZUMO1 [6], [16]. Therefore, we tried to search for an IZUMO1-interacting protein in this experiment and identified ACE3 as an IZUMO1-interacting protein. The previously-reported mRNA sequence for *Ace3* was slightly different to the one described in this paper. In our experiment, the initial methionine started 56-bp upstream of the previously-reported *Ace3* and our sequence indicated the existence of an intron in the previously-reported first exon (Figure 2A). The expression pattern was also different from the previous report and in our hands, *Ace3* was exclusively expressed in sperm. Therefore we referred to our ACE3 as tACE3 in this manuscript.

The tACE is known to regulate sperm migrating ability into oviduct and binding ability to zona pellucida of eggs [8], [9]. tACE may not able to interact with IZUMO1 because their localizations are topologically separated each other in sperm before acrosome reaction (plasma membrane for tACE and acrosomal membrane for IZUMO1). However, present experiment revealed that there is another ACE-related protein on sperm and that this newly found tACE3 is indicated to interact with IZUMO1. It is reported that IZUMO1 protein forms a protein complex containing several large proteins under mildly denaturing conditions on SDS-PAGE [5]. ACE2 is also known to exist in testis, but the expression site for ACE2 is limited to Leydig cells in testis [17]. Moreover, no impairment of fertilizing ability of *Ace2*-disrupted mice is reported [18]. Thus the only possible ACE homologue that can interact to IZUMO1 is the tACE3.

In mouse, rat, cow, and dog, the tACE3 contains an HQMGH motif. However, the Glu included in this motif was substituted by Gln in all of these species. Since the Glu residue is 100% conserved in all other ACE family members and is considered to be critical for their catalytic activity, tACE3 may not have ACE protease activity [7]. In 2005, Kondoh et al. reported that tACE has both peptidase activity (to cleave angiotensinogen) and glycosylphosphatidylinositol (GPI)-releasing activity, and that this secondary activity is crucial for sperm fertilizing ability [19], [20]. Another paper also suggests that sACE may possess GPI-ase activity [21]. However, Fuchs S et al. argues against this conclusion by providing evidence that sperm from mutant ACE knock-in male mice, in which HEMGH was altered to KEMGK to abrogated the peptidase activity, the male mice lost their ZP binding ability [22]. Since we demonstrated that tACE3, which is predicted to naturally lack peptidase activity, could be an IZUMO1-interacting protein, analysis of the phenotype of the tACE3 disruption was expected to provide important information regarding the mechanism of fertilization.

However, the disruption of *Ace3* resulted in no demonstrable defect in fertilizing ability. Thus the importance of GPI-ase activity for tACE protein family function requires further clarification. The question as to whether IZUMO1 is functioning as a sole essential factor in fusion, or rather is participating as a part of protein complex [5], remains unanswered. The only phenotype found in *Ace3*-deficient sperm is that the localization of IZUMO1 extended to a broader area than in wild-type sperm. This difference may be caused by the loss of interaction of tACE3 with IZUMO1 in sperm, but the aberrant distribution caused by *Ace3* disruption was not critical enough to affect the sperm fertilizing ability.

Considering the published data together with phenotype of *Ace3*-disrupted mice described in this paper, it becomes clear that among ACEs, the essential ACE for sperm fertilizing ability is solely the tACE. Further study is required to elucidate the mechanism of fertilization, but the results presented in this paper help to narrow the path leading to the answer.

## Materials and Methods

### Animals

IZUMO1-null mice were prepared as indicated in our previous paper [4]. BDF1 male and female mice were purchased from CLEA Japan. All of the experiments were performed with the approval of the Animal Care and Use Committee of Osaka University (permit number: BikenH20-09-1).

### Identification of ACE3

We previously established His-tagged mouse IZUMO1 transgenic mouse lines with *Izumo1* ^−/−^ background to evaluate the requirement for IZUMO1 in fertility [10]. Utilizing these male mice, mature sperm were collected from cauda epididymis, and solubilized with 1% Brij 97, 150 mM NaCl, 50 mM Tris-HCl (pH 8.0) and 1% protease inhibitor cocktail (Nakalai tesque, Kyoto, Japan). After centrifugation at 15,000 g for 30 min at 4°C, the insoluble fraction was removed and the supernatant incubated with anti-His microbeads (μMACS anti-His microbeads; Miltenyi Biotec, Bergisch Gladbach, Germany). The samples were prepared following the manufacturer's instructions. After separation by SDS-PAGE, protein gels were stained with the silver staining kit (Nakalai tesque, Kyoto, Japan) to visualize protein bands. IZUMO1-interacting protein was determined by LC MS/MS (Figure 1A). Some peptide sequences (Figure 1B, shown in red) were analyzed and identified as tACE3 protein (NCBI accession number: XP_110936), whose function was not known [7]. To confirm the DNA sequence, we amplified (RT-PCR) *Ace3* from mouse testis RNA as a template using primers derived from this sequence. The polyclonal antibodies against mouse tACE3 were produced by immunizing KLH-conjugated specific peptides to rabbits (Figure 1B, shown in blue).

### Western blotting

Proteins from various tissues were solubilized with 1% Triton X-100, 150 mM NaCl, 50 mM Tris-HCl (pH 8.0) and 1% protease inhibitor cocktail (Nakalai tesque, Kyoto, Japan), and were centrifuged at 15,000 g for 30 min at 4°C. The supernatants were denatured by boiling for 5 min in the presence of 1% SDS with or without 6% 2-mercaptoethanol, separated by SDS-PAGE, and transferred onto Immobilon-P membranes (Millipore, MA, USA). After blocking with 10% skim milk, the blots were incubated with primary antibodies for 2 h and then incubated with horseradish peroxidase-conjugated secondary antibodies for 1 h. Immunoreactive proteins were detected by an ECL western blotting detection kit (GE Healthcare, Little Chalfont, England).

### Immunoprecipitation

Sperm supernatants (1 ml, 1.5 mg protein per ml) solubilized with 1% Brij 97, 150 mM NaCl, 50 mM Tris-HCl (pH 8.0) and 1% protease inhibitor cocktail were rotated overnight at 4°C with rabbit polyclonal tACE3 and rat monoclonal IZUMO1 antibodies (10 µg/ml). Mixtures with protein G MicroBeads (50 µl, 1 h, Miltenyi Biotec, Auburn, CA) were loaded on MACS columns (Miltenyi Biotec, Bergisch Gladbach, Germany), washed (3×200 µl of 25 mM Tris (pH 8.0)/1.0 M NaCl/1% Brij 97), and eluted with gel loading buffer [50 mM Tris (pH 6.8)/100 mM DTT/2% (wt/vol) SDS/0.1% bromophenol blue/10% glycerol]. Eluates were separated by denaturing SDS/PAGE, and were subjected to western blotting. Immunodetection was done with 1 µg/ml of rabbit anti-tACE3 and rat anti-IZUMO1 antibodies. Secondary antibodies were horseradish-peroxidase-conjugated goat anti-rabbit or goat anti-rat IgGs (1∶10,000, The Jackson Laboratory, Maine, USA). Chemiluminescent detection was performed with the enhanced chemiluminescence (ECL) kit (GE Healthcare, Little Chalfont, England), as instructed.

### Immunofluorescence analysis

The spermatozoa prepared from cauda epididymis were washed twice with PBS, spotted onto slides, air dried, fixed with ice-cold 100% EtOH for 5 min and rinsed briefly in PBS. Nonspecific protein binding sites were blocked with 10% (wt/vol) Block Ace (Yukijirushi Co., Tokyo, Japan) and 10% (vol/vol) goat serum–PBS at room temperature for 30 min. The slides were then incubated with 4 µg/ml anti-tACE3 or anti-IZUMO1 antibodies at 4°C overnight. After being washed with PBS, the slides were incubated in the dark with Alexa Fluor 488 or 546-conjugated anti-rabbit or anti–rat IgGs, respectively (Invitrogen, California, USA) at 37°C for 30 min in PBS containing 10% Block Ace and 10% goat serum. After being washed, the slides were mounted in PBS. The stained cells were observed under a fluorescence microscope.

### Generation of Ace3 knockout mice

A targeting vector was constructed using pNT1.1 containing Neo-resistance gene (Neo^r^) as a positive selection marker and herpes simplex virus thymidine kinase gene (tk) as a negative selection marker. A 1.4-kb *Not* I-*Xho* I fragment and a 6.6-kb *Asc* I-*Pac* I fragment were inserted as a short and long arm, respectively. Embryonic stem cells derived from 129/Sv were electroporated with *Pme* I digested linearized DNA. Of 352 G418-resistant clones, four had undergone homologous recombination correctly. Two targeted cell lines were injected into C57BL/6 blastocysts, resulting in the birth of male chimeric mice. These mice were then crossed with C57BL/6 to obtain heterozygous mutants. Mice used in the study were the offspring of crosses between F1 and/or F2 generations. The PCR primer used for genotyping were as follows: 5′-CTCTTCCAAGTTCTATGCAACGGAGTCCTT-3′ and 5′-GCTTGCCGAATATCATGGTGGAAAATGGCC-3′ for the short arm side of mutant allele, 5′-CTCTTCCAAGTTCTATGCAACGGAGTCCTT-3′ and 5′-CCTGAGCCTCGCTGTAGAAATCATCTGCAA-3′ for the short arm side of wild-type allele, 5′-TTCTCGGTGTTCTGCGTGACTATCCGAATG-3′ and 5′-TCTGTTGTGCCCAGTCATAGCCGAATAGCC-3′ for the long arm side of mutant allele, 5′-TTCTCGGTGTTCTGCGTGACTATCCGAATG-3′ and 5′-CCTGTTAGGCATTGCCCTGGAGAAG-3′ for the long arm side of wild-type allele. The *Ace3* disrupted mouse line was submitted to RIKEN BioResource Center and is available to the scientific community.

### *In vitro* fertilization

Mouse sperm were collected from cauda epididymides and capacitated *in vitro* for 2 h in 200 µl drops of TYH medium covered with paraffin oil. BDF1 female mice (>8 weeks old) were superovulated with an injection of 5 IU of hCG (human chorionic gonadotropin) 48 h after a 5 IU injection of eCG (equine chorionic gonadotropin). The eggs were collected from the oviduct 14 h after the hCG injection. Eggs were placed in a 200 µl drop of TYH medium. For preparation of cumulus free eggs, eggs were freed from cumulus cells with 0.01% (w/v) hyaluronidase in advance. These eggs were incubated with 2×10^5^ *Ace3* ^+/−^ or *Ace3* ^−/−^ sperm/ml incubated for 2 h at 37°C in 5% CO_2_, and unbound sperm were washed away. Eggs were observed 12 h after insemination for 2-cell formation under a Hoffman modulation contrast microscope.

### Sperm-egg fusion assay

Mouse sperm and eggs were prepared as above. After being freed from cumulus cells with 0.01% (w/v) hyaluronidase, the zona pellucida was removed from mouse eggs using a piezo-manipulator as previously reported [23]. The zona-free eggs were pre-loaded with Hoechst 33342 by incubating them with 1 µg/ml of the dye in TYH for 10 min. After washing, the eggs were incubated with 2×10^5^ sperm/ml incubated for 30 min at 37°C in 5% CO2, and unbound sperm were washed away. The eggs were observed under a fluorescence microscope (UV excitation light) after fixing with 0.25% glutaraldehyde. This procedure enabled staining of only fused sperm nucleus by transferring the dye into sperm after membrane fusion.
